# A systematic review and meta-analysis comparing complications following total hip arthroplasty for systemic lupus erythematosus versus for non-systemic lupus erythematosus

**DOI:** 10.1186/s13018-022-03075-8

**Published:** 2022-04-12

**Authors:** Yiwei Huang, Danni Guan, Yijin Li, Jiahao Li, Yirong Zeng

**Affiliations:** 1 The First Clinical Medical School, Guangzhou University of Chinese Medicine, Jichang Road 12#, District Baiyun, Guangzhou, Guangdong China; 2 Science and Technology Innovation Center, Guangzhou University of Chinese Medicine, Jichang Road 12#, District Baiyun, Guangzhou, Guangdong China; 3 Department of Orthopaedics, The First Affiliated Hospital of Guangzhou University of Chinese Medicine, Jichang Road 16#, District 22 Baiyun, Guangzhou, 510405 Guangdong China

**Keywords:** Systemic lupus erythematosus, Total hip arthroplasty, Meta-analysis

## Abstract

**Background:**

Osteonecrosis of the femoral head is one of the most severe complications in systemic lupus erythematosus (SLE) patients. Total hip arthroplasty (THA) is an effective treatment for femoral head necrosis. However, there is no consensus on the specific effect of THA on SLE patients. The objective of the present study was to review the current evidence regarding rates of THA complications and postoperative function in systemic lupus erythematosus.

**Methods:**

Two independent reviewers searched PubMed, Cochrane Library, and EMBASE from January 1, 2000, to December 29, 2021. The primary outcomes were postoperative complications, including deep vein thrombosis (DVT), hematoma, wound infection, dislocation, periprosthetic fracture, revision, mortality.

**Results:**

A total of 179 articles yielded 28 studies eligible for inclusion with 10 studies used for meta-analysis. This study found a statistically significant difference in DVT, dislocation, wound infection, periprosthetic fracture, and revision.

**Conclusions:**

This meta-analysis shows that SLE patients with THA are at an increased risk of DVT, wound infection, dislocation, periprosthetic fracture, revision, periprosthetic joint infection, following THA in comparison with non-SLE patients with THA. There was no adequate evidence to support the notion that the risk of seroma or hematoma following THA is increased in SLE. Also, there was no significant difference in HHS scores between SLE patients and non-SLE patients after THA.

**Supplementary Information:**

The online version contains supplementary material available at 10.1186/s13018-022-03075-8.

## Background

Systemic lupus erythematosus (SLE), a prototype chronic autoimmune disease, can lead to a variety of various complications, including osteonecrosis of the femoral head (ONFH) [1]. Cortisol hormones are the basis of SLE therapy, but excessive use of cortisol hormone is an essential causative of femoral head necrosis. Hussein reported that about 10% of patients with SLE would progress ONFH [2]. The general prevalence of SLE in the population is estimated to be about 1/1000. However, due to racial and gender differences, the incidence rate of women is about ten times higher than that of men, especially women of childbearing age [3]. Besides, using cortisol hormone can improve mortality outcomes and is associated with excess risk for avascular necroses, osteoporosis, and pathological fractures [4].

With the improvement of the life span of patients with SLE, effective treatment measures are more urgent [5]. THA is still one of the best treatments for advanced ONFH [6]. However, long-term use of cortisol hormone in patients with SLE increases the risk of surgery. In patients with SLE, disease activity and infection are the two leading causes of postoperative death [3]. Some studies have reported several complications of total hip replacement in patients with SLE and postoperative Harris hip scores (HHS) and other functional scoring results [7–16]. At present, there is no clear consensus on the impact of complications after THA in patients with SLE. Patients with SLE undergoing THA are widely considered to have an increased risk of postoperative complications [10, 17]. However, some people reported no significant increase in the incidence of complications [3, 18]. Understanding the incidence of complications (risk–benefit) is crucial for patients with SLE and surgeons when considering surgery [19].

The purpose of this systematic review was to determine the overall recovery of hip joint function and incidence of complications of total hip arthroplasty in patients with SLE over the past 22 years, including specific rates of deep vein thrombosis (DVT), hematoma, wound infection, dislocation, periprosthetic fracture, revision and mortality.

## Methods

### Search strategy

The guidelines for systematic reviews of prevalence studies by the Preferred Reporting Items for Systematic Reviews and Meta-Analyses (PRISMA) 2020 recommendations were followed [20]. Subsequently, we searched the following databases: PubMed, Cochrane Library, and EMBASE, from January 1, 2020, to December 29, 2021. The search strategy followed Medical Subject Headings combination with terms (Additional file 1) for (1) Lupus Erythematosus, Systemic; (2) Arthroplasty, Replacement, Hip, but only included articles in English.

### Study selection and data extraction

Two independent review authors (Danni Guan and Yijin Li) screened all titles and abstracts using clearly defined inclusion and exclusion criteria. Only English-language publications on patients who reported complications of total hip replacement in patients with systemic lupus erythematosus or some postoperative Harris hip scores (HHS) were included for further examination.

According to the PICOS order, the study included in our meta-analysis had to meet all of the following requirements: (1) Population: The experimental group was diagnosed with SLE patients, and the control group was non-SLE patients; (2) Intervention: All patients underwent primary THA; (3) Outcomes: the article should include complications or some hips functional scores after total hip arthroplasty in all patients, such as HHS and Western Ontario and McMaster University (WOMAC) osteoarthritis index. Studies will be excluded: (1) non-research papers (2) systematic reviews; (3) animal models; (4) case reports or case series.

According to the Cochrane guidelines, two independent review authors initially screened relevant articles based on title and abstract. Relevant data extracted included publication information (author, study design, and year), patient baseline characteristics (gender and age), and outcome data (DVT, hematoma, wound infection, dislocation, periprosthetic fracture, revision, periprosthetic joint infection, mortality).

### Quality assessment

According to the Newcastle–Ottawa scale (NOS), all articles were used to evaluate the quality of any methods included in the study [21]. This scale contains eight items, divided into three dimensions: selection, comparability, outcome measurement. Two investigators independently assessed all studies, and if it gets inconsistent scores, the study will be resolved through discussion by a third reviewer.

### Statistical analysis

All extracted statistics analysis and figure production were performed with the Review Manager (version 5.4 for Windows). To assess the dichotomous variables in the study (such as postoperative complications), we usually choose the odds ratio and the related 95% confidence interval for measurement. Individual complication rates were calculated by dividing the number of patients with SLE with complications after THA by the total number of patients undergoing THA. The overall complication rate was calculated by adding the dislocation rates, DVT, infection, aseptic loosening, and revision for each study [19]. Then, we involved studies that provided a complete mean and standard deviation. Continuous variables such as HSS are evaluated by using mean difference (MD) or standard mean difference (SMD). Heterogeneity between studies was assessed by *I*^2^ and *Q* tests. When *I*^2^ ≥ 50%, the random effect model is used instead of the fixed-effect model [22]. The overall effect of each study was shown by forest map, and the Deeks' funnel plot evaluated the publication bias.

## Result

### Study selection

**Fig. 1:**
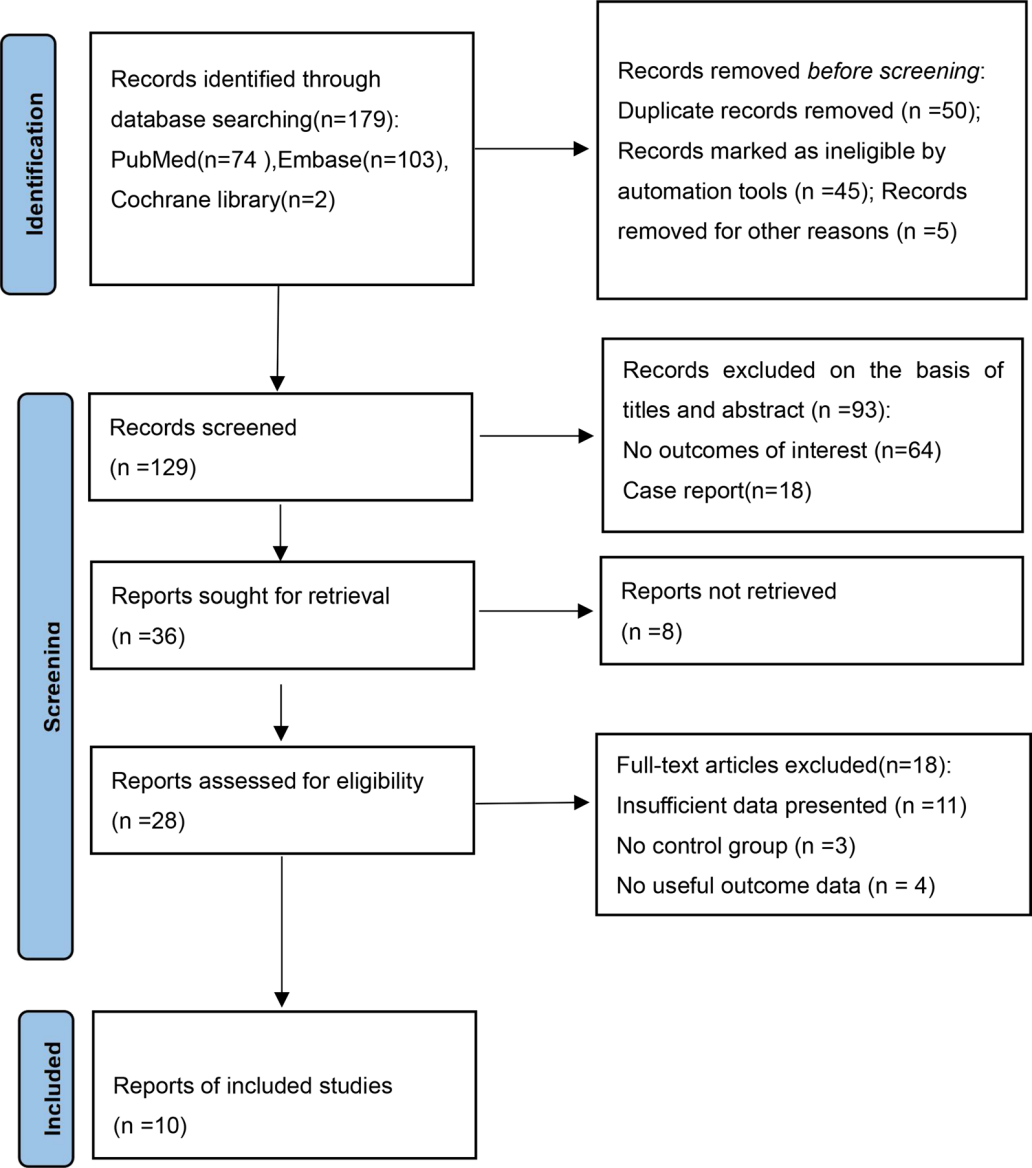
The search strategy flowchart of study selection

Through the above search strategy, 179 related papers were selected from three databases. After deleting the duplicate literature, 129 articles remained. By reading the title and abstract, 93 studies that did not meet our requirements were deleted; the remaining 36 articles were further read in full text. Finally, after reading the complete text, 28 articles were included in the systematic review, and ten articles were included in the meta-analysis. The exclusion reasons include insufficient data, no control group, and no effective date. The complete studies screening process is shown in the PRISMA flowchart in Fig. 1.

### Study characteristics and quality assessment

Demographics and clinical outcomes are summarized in Tables 1 Tables 2. Among the ten screened citations, seven were the case–control studies [7, 11, 14, 15, 17, 23, 24], two were retrospective [18, 25], and one was cohort studies a study [10]. A total of 29,573 hips in the SLE group were studied compared to 4,013,027 hips in the non-SLE group. Among the ten articles, eight ultimately recorded the number and types of complications, with common reasons such as DVT, hematoma, wound infection, dislocation, periprosthetic fracture, and revision, but only three studies recorded the mean value standard deviation of preoperative and postoperative HSS. The quality of 10 studies included in the meta-analysis assessed with the Newcastle–Ottawa scale (Tables 3) ranged from five to nine.

**Table 1:** Demographics of the included studies

Author	Years	Design	Case/Hips	Mean age	Female sex (%)	Follow up (year)	Outcome measures
Aziz [10]	2020	Cohort study	15,883	56 ± 0.4	13,557 (87)	9	Complication
Cai [14]	2021	Case–control study	45	40.78	40 (88.67)	6	Complication, HHS
Chen [11]	2021	Case–control study	617	38.7 ± 12.7	503 (81.5)	17	Complication
Chen [7]	2019	Case–control study	557	41	**–**	0.25	Complication
Gu [15]	2021	Case–control study	92	39.9 ± 13.6	74 (80.4)	13	Complication, HHS, PCS, MCS
Issa [25]	2013	Retrospective study	40/60	42	**–**	7	Complication, HHS
Merayo-Chalico [23]	2017	Case–control study	58	34.4 ± 1.05	**–**	18	Complication
Roberts [24]	2016	Case–control study	58	52.0	52 (89.6)	4	Complication, WOMAC
Schnaser [17]	2016	Case–control study	12,555	53	10,995 (88)	9	Complication
Woo [18]	2014	Retrospective study	13/19	41.3 ± 12.5	12 (92)	8	Complication, HHS

HHS, Harris hip scores; WOMAC, Western Ontario and McMaster University osteoarthritis index; PCS, Physical Component Summary Scale score; MCS, Mental Component Summary Scale score

**Table 2:** Summary of clinic outcomes for each study

Author	Years	SLE		Non-SLE	
		event	Total	event	Total
Aziz [10]	2020	Acute renal failure 196 Death 28 Myocardial infarction 0 Pneumonia 178 Stroke 13 Any major complication 496 DVT 39 Hip dislocation 42 General complication 813 Seroma or hematoma 213 Wound infection 64 Any minor complication 842 Pulmonary embolism 34	15,583	Acute renal failure 30,380 Death 2648 Myocardial infarction 1644 Pneumonia 18,036 Stroke 1498 Any major complication 56,218 DVT 2825 Hip dislocation 2938 General complication 99,025 Seroma or hematoma 23,749 Wound infection 5434 Any minor complication 10,125 Pulmonary embolism 3961	1,990,939
Cai [14]	2021	Ecchymosis 2 Wound infection 2 Wound fat liquefaction 2 Wound swelling 0 Hypocalcemia 1 Hypoproteinemia 8 Hypokalemia 6 Anemia 7 Vomiting 2 Fever 2 Pulmonary infection 1 PE 0 SF-12 48.91 Dislocation 4 Infection 0 Periprosthetic fracture 0 Revision 0 Aseptic loosening 0	45	Ecchymosis 0 Wound infection 0 Wound fat liquefaction0 Wound swelling 6 Hypocalcemia 0 Hypoproteinemia 4 Hypokalemia 1 Anemia 2 Vomiting0 Fever 0 Pulmonary infection 1 PE 0 SF-12 52.38 Dislocation 2 Infection 0 Periprosthetic fracture 0 Revision 0 Aseptic loosening 0	45
Chen [11]	2021	superficial wound infection 44 PJI 22 Pneumonia 0 Urinary tract infection 0 Pulmonary embolism 0 DVT 1 In-hospital mortality 0	325	superficial wound infection 20 PJI 9 Pneumonia 0 Urinary tract infection 7 Pulmonary embolism 0 DVT 0 In-hospital mortality 0	325
Chen [7]	2019	Fracture of the lower limb 47	557	Fracture of the lower limb 127	2885
Gu [15]	2021	Hematoma formation 9 Superficial infection 4 New symptomatic DVT 9 Periprosthetic fracture 0 Total hip procedures 20	92	Hematoma formation 2 Superficial infection 0 New symptomatic DVT 4 Periprosthetic fracture 1 Total hip procedures 7	92
Issa [22]	2017	Surgical site hematoma 4 Bleeding secondary to over anticoagulation 1 Urinary tract infection 3 Transfusion requirement 8 Soft tissue infection 1 Bacteremia 0	58	Surgical site hematoma 0 Bleeding secondary to over anticoagulation 0 Urinary tract infection 1 Transfusion requirement 2 Soft tissue infection 0 Bacteremia 0	58
Merayo-Chalico [23]	2017	Acute renal insufficiency 5 Arrhythmia 1 DVT 3 Falls 6 Postoperative fracture 2 Dislocation 5 Additional surgery 4 Superficial surgical site infection 4 Excessive surgical site drainage 3 Surgical site ecchymosis 0 Surgical site erythema 3 Spinal headache 2 Delayed wound healing 0	58	superficial wound infection 20 PJI 9 Pneumonia 0 Urinary tract infection 7 Pulmonary embolism 0 DVT 0 In-hospital mortality 0	116
Roberts [24]	2016	Cardiac 103 Peripheral vascular 19 Respiratory 85 Gastrointestinal 42 Genitourinary 61 Central nervous system 14 Postoperative anemia 3264 Deep vein thrombosis 108 Pulmonary embolism 34 Hematoma/seroma 163 Wound dehiscence 0 Infection 34 Prosthetic hip dislocation 35 Periprosthetic fractures 36 Mortality 20	12,555	Cardiac 16,624 Peripheral vascular 1510 Respiratory 12,619 Gastrointestinal 13,768 Genitourinary 14,681 Central nervous system 2952 Postoperative anemia 444,150 Deep vein thrombosis 6022 Pulmonary embolism 3961 Hematoma/seroma 20,997 Wound dehiscence 249 Infection 2512 Prosthetic hip dislocation 2822 Periprosthetic fractures 1860 Mortality 2208	2,018,567

**Table 3:** Quality assessment for the studies included in the meta-analysis (NOS)

Author	Selection	Comparability	Exposure or outcome	Total score
Aziz[10]	★★★★	★★	★★★	9
Cai[14]	★★	★	★★	6
Chen[11]	★★★	★★	★★★	8
Chen[7]	★★★★	★	★★	7
Gu[15]	★★★	★★	★★	7
Issa[25]	★★	★	★★	5
Merayo-Chalico[23]	★★★	★	★★	7
Roberts[24]	★★★	★	★★★	7
Schnaser[17]	★★★★	★★	★★★	9
Woo[18]	★★	★	★★	5

NOS Newcastle–Ottawa scale

### Complication

#### Wound infection

**Fig. 2:**
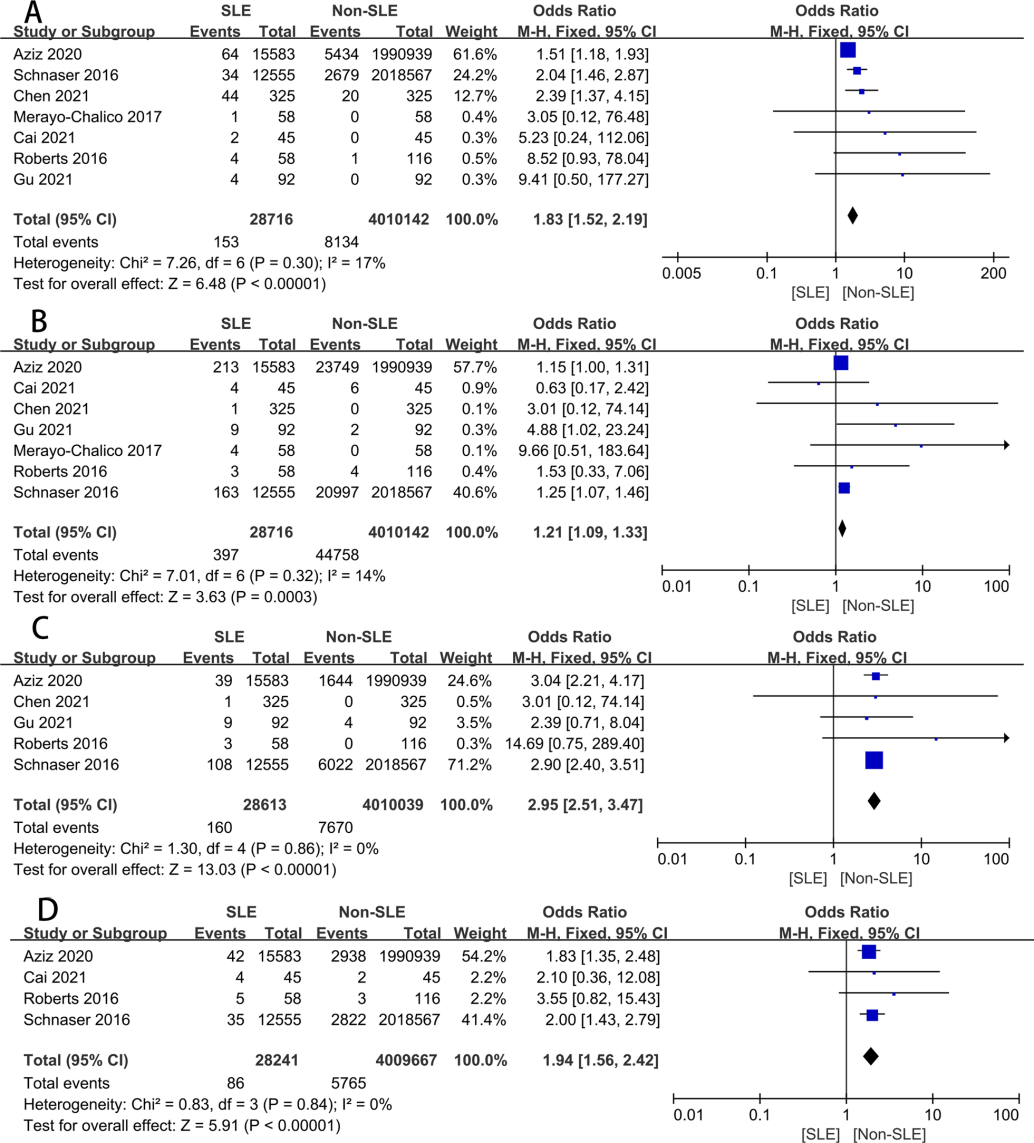
Forest plots for the wound infection (**A**), seroma or hematoma (**B**), DVT (**C**), dislocation (**D**); DVT, deep vein thrombosis

**Fig. 3:**
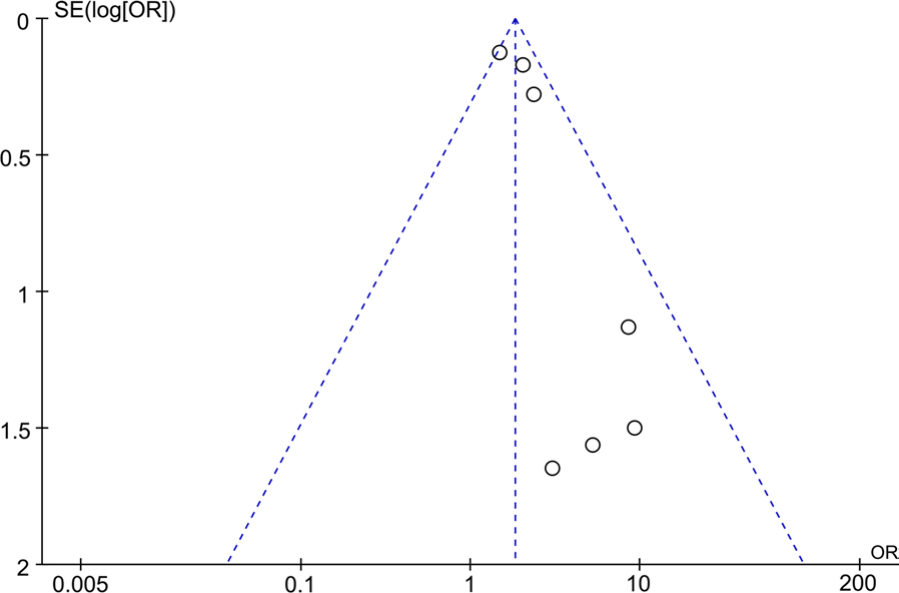
Funnel plots for reporting the wound infection

The wound infection was used in seven studies [10, 11, 14, 15, 17, 23, 24], and the results in the meta-analysis showed significant differences. According to Fig. 2A, the wound infection (OR 1.83, 95% CI 1.52–2.19, *P* = 0.30, *I*^2^ = 17%; Fig. 2A) was 1.83 times higher in the SLE group than in the Non-SLE group. In this meta-analysis, we chose a fixed effect model because the results of the heterogeneity analysis (*P* = 0.30, *I*^2^ = 17%) indicated essentially no heterogeneity. Furthermore, our study shows that no literature will significantly interfere with the results by sensitivity analysis, which shows that this study has good accuracy and stability. The pooled information was shown in our funnel plot (Fig. 3). Due to differences in infection and preoperative antibiotic regimens (some centers give up antibiotics completely) and sample size, there will be some differences in the meta-analysis. However, it is also within the acceptable range. Aziz [10] et al. searched the National Inpatient Sample (NIS) database for SLE and Non-SLE patients who underwent primary THA from 2000 through 2009. In the NIS database, the discharge information of 20% stratified sampling of American hospitals and the patient information of all payers were counted. It is reported that this sampling method collected 97% of hospital discharge cases in the USA. Aziz and colleagues found that patients with SLE had an OR of 1.51 (95% CI 1.18–1.93) for wound infection following THA, relative to patients. It is worth noting that the result is consistent with the conclusion of Schanzer et al. [17]

#### Seroma or hematoma

Seven studies [10, 11, 14, 15, 17, 23, 24] compared the rates of seroma or hematoma in patients with SLE versus non-SLE of THA. Meta-analysis of these seven studies, all of which reported unadjusted comparative data, revealed a slightly increased risk of seroma or hematoma of THA in patients with SLE relative to those with non-SLE (OR 1.21 95%, CI 1.09–1.33, *P* = 0.32, I2 = 14%) (Fig. 2B). There is no heterogeneity in our meta-analysis.

#### DVT

Five studies [10, 11, 15, 17, 24] addressed DVT. Similarly, fixed-effects models were used to calculate because no evidence of heterogeneity was found in the study (OR 2.95, 95% CI 2.51–3.47, *P* = 0.86, *I*^2^ = 0%). The forest plot showed that the rate of DVT was 2.95 times higher in the SLE group than in the non-SLE group (Fig. 2C).

#### Dislocation

Four studies [10, 14, 17, 24] provided sufficient information in this meta-analysis. Meta-analysis of these 4 studies shows an increased risk of hip dislocation of THA in patients with SLE than those with Non-SLE (OR 1.94, 95%, CI 1.56–2.42, *P* = 0.84, *I*^2^ = 0%) (Fig. 2D).

#### Periprosthetic fracture

**Fig. 4:**
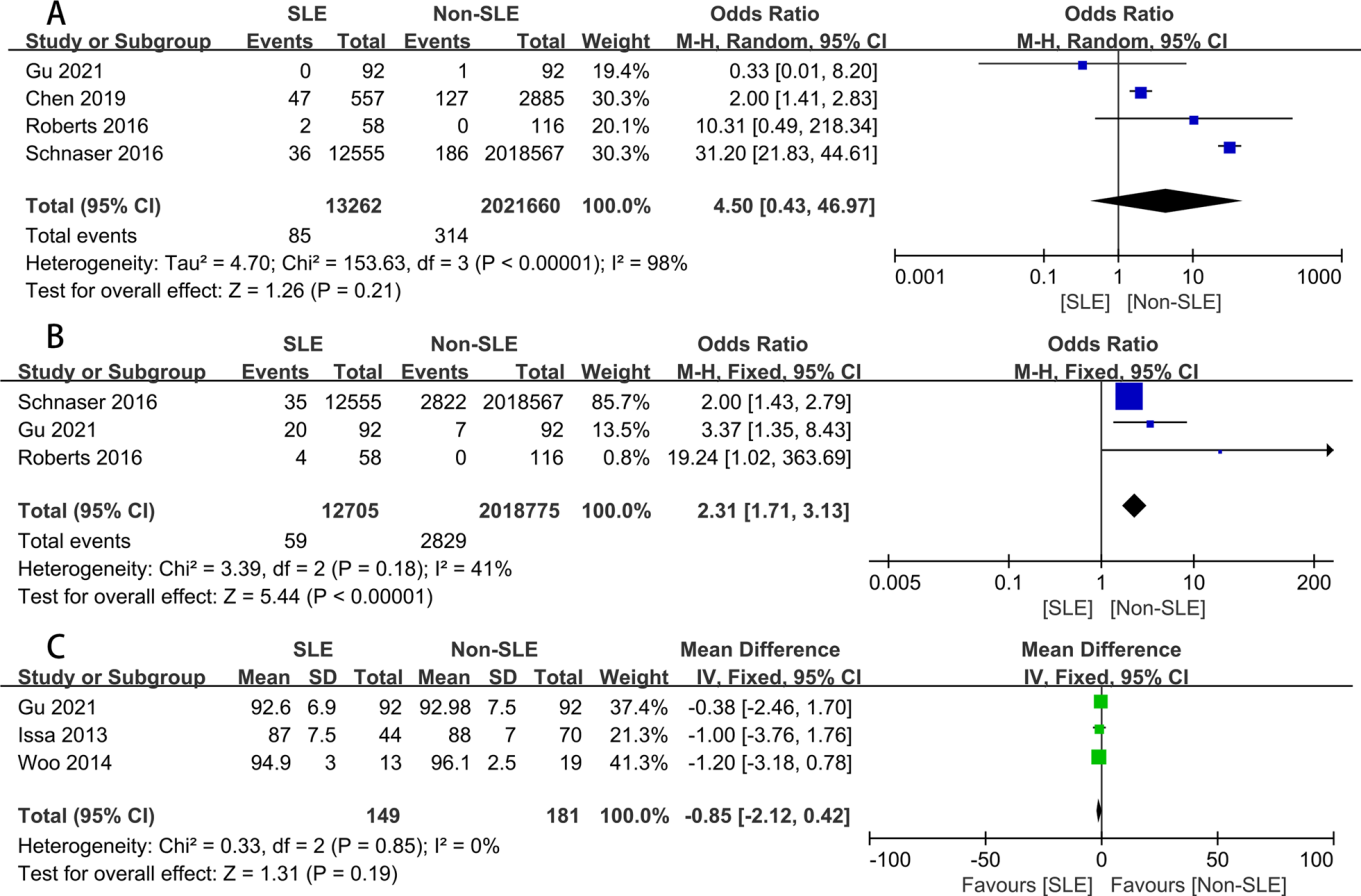
Forest plots for the Periprosthetic fracture (**A**), revision (**B**), HSS (**C**); HSS, Harris hip scores

Four studies [7, 15, 17, 24] reported periprosthetic fracture on events following THA. After the heterogeneity test (OR 4.50, 95% CI 0.43–46.97, *I*^2^ = 98% > 50%, *P* < 0.1), it shows that the heterogeneity between the documents selected in this study is statistically significant, so we chose a random effect model, and it is necessary to find the heterogeneity (Fig. 4A). We discovered that Schnaser 2016 had a significant impact on heterogeneity by analyzing 4 literature of sensitivity, After removing the study, the results showed no heterogeneity in the remaining 3 kinds of literature (*I*^2^ = 14% < 50%, *P* = 0.31 > 0.1). After exclusion, the forest plot showed that the rate of Periprosthetic fracture was 2.01 times higher in the SLE group than in the non-SLE group (OR 2.01, 95% CI 1.43–2.82). However, considering that Schnaser 2016 study is a stratified sampling of NIS in the American hospital system, with a large sample size, long follow-up time, high research quality, and low-risk bias, this study is still retained.

#### Revision

Three [15, 17, 24]studies compared the revision rates. Meta-analysis of these 3 studies reveals a slightly increased risk of revision of THA in patients with SLE relative to those with non-SLE (OR2.31, 95% CI 1.71–3.13, *P* = 0.18, *I*^2^ = 41%) (Fig. 4B). There is no heterogeneity in our meta-analysis.

#### HSS

The HSS score was used in 3 studies [15, 18, 25], and the results of postoperative with SLE in meta-analysis showed no significant differences after THA (MD − 0.85, 95% CI − 2.12 to 0.42, *P* = 0.85, *I*^2^ = 0%; Fig. 4C), compared to preoperative result. Fixed effects models were used to calculate because no evidence of heterogeneity was found in the study (*P* = 0.85, *I*^2^ = 0%).

#### Other complication

In addition to comparing the above complications, the meta-analysis also recorded the occurrence of other complications, such as pneumonia, pulmonary embolism, urinary tract infection, and anemia. Only Aziz [10] and Schnaser [17] said the patients with SLE after THA number of mortality and pulmonary embolism in the 10 studies. Chen [11], Javier [23] and Schnaser [17] reported that patients with SLE after THA had complications of urinary tract infection. In the study reported by Chen [11], the incidence rate of PJI after THA in the SLE group and the non-SLE group is higher than in other research. The specific number of complications can be seen in Table 2.

## Discussion

SLE is a complex disease with a multifactorial etiology. Long-term use of cortisol hormone is a significant risk factor for osteonecrosis. Other risk factors, such as arthritis, neuropsychiatric manifestations of SLE, vasculitis, hypertension, serositis, and renal disease, may be moderately associated with ON, and a SLEDAI score > 8 may be strongly associated with ON, but this is still uncertain due to the very low quality of evidence [26]. According to Abu-Shakra et al. [27], patients with systemic lupus erythematosus have an increased risk of osteonecrosis. On the contrary, Fein et al. [28], Roberts et al. [24] determined ON and adverse events were not related to steroid use.

With the increase in life expectancy, the demand for THA in patients with SLE also increases, doubling from 0.17/100,000 in 1991 to 0.38/100,000 in 2005. In addition, the main indications in this series are in nearly a quarter of cases [29]. Chen et al. [30] report a 190% increase in THA for ON in patients with SLE from 2007 to 2015. However, there is no accurate conclusion on the specific effect of THA on SLE patients.

To our knowledge, this is the first systematic assessment of complications after total hip arthroplasty (THA) in SLE and non-SLE patients. Our meta-analysis of 10 studies found reliable evidence for increased risk of DVT, dislocation, wound infection, periprosthetic fracture, and revision. There was no obvious evidence for increased risk of seroma or hematoma, following THA, in patients with SLE versus non-SLE patients. We also found no significant difference in postoperative HHS scores of patients with SLE and non-SLE patients.

Our studies suggest an increased risk of dislocation and periprosthetic fracture among SLE patients compared with the non-SLE patients. Schnaser et al. [17] reported that Patients with SLE often have compliant soft tissue and an excellent range of motion preoperatively, which may be a plausible explanation. As for periprosthetic fracture, another potential cause of THA in patients with SLE is femoral neck fractures secondary to osteoporosis. Schnaser et al. [17] show that SLE patients appear to have a significant increase in perioperative fracture when compared with patients with OA. SLE patients tend to be osteopenic, and this could account for the increased rate of fracture seen. And Roberts et al. [24] also reported that the SLE cohort experienced a higher rate of falls than the osteoarthritis controls. Roberts et al. also explain that SLE patients are prone to fall for several reasons, including multi-drug combination and use of immunosuppressants, lack of vitamin D, and weaker functional status and strength than non-SLE patients.

It is worth noting that the relationship between SLE patients undergoing THA and wound infection is not apparent in some study. Woo et al. [18] reported one revision for osteolysis at nine-year follow-up in 19 arthroplasties of SLE patients who not experienced other postoperative complications. Chen et al. [30] reported that 244 Patients with SLE undergoing THA for osteonecrosis experienced lower rates of infection (OR 0.3; 95% CI 0.2–0.5) and revision (OR 0.71; 95% CI 0.6–0.9), but a higher rate of medical complications (OR 1.22; 95% CI 1.0–1.5) within 90 days compared to patients undergoing THA for non-SLE-related osteonecrosis diagnoses. However, some cohort studies have found opposite results. It makes intuitive sense that the risk of infection following THA would increase in patients due to differences in the pathogenesis and medical management of these conditions. Roberts et al. [24] reported 4 superficial wound infections following 58 THAs with SLE patients, but 1 superficial wound infection and 2 delayed wound healing in a cohort of 116 THA with Non-SLE patients. More recently, Chen et al. [11] have reported an infection rate of 13.6% following 325 arthroplasties with SLE patients and an infection rate of 6.1% following 325 arthroplasties with Non-SLE patients. Osteonecrosis was related to younger age, corticosteroid use, excessive drinking, smoking, HIV, sickle cell, and organ transplantation [31]. Some reasons could explain this discrepancy. For example, Kang described the use of a more aggressive prophylactic antibiotic regimen in THA with SLE patients, and the previous study of THA in patients with systemic lupus erythematosus was focused on patients with inactive or slightly active diseases [32].

Notably, our meta-analysis showed that the wound infection rate in the SLE group was 1.85 times higher than that in the non-SLE group. Our review revealed fair evidence to support the notion of an increased risk of infection following THA in patients with SLE versus Non-SLE. Six of the seven pieces of literature we included suggested that SLE patients had an increased risk of infection after THA, and two had a lower risk of bias.

As for PJI, in our meta-analysis, one article recorded the incidence rate of PJI with 325 SLE patients and 325 non-SLE Patients. Unlike what we expected, in Chen's study, by controlling for other confounding factors, it was found that the disease status of SLE did not significantly increase the risk of perioperative wound infection or PJI [11].

Although we did not analyze the specific data of blood transfusion in SLE patients undergoing THA surgery, we also noticed that many articles mentioned anemia after SLE surgery. Chen et al. [30] found that the disease status of SLE did not significantly increase the risk of perioperative wound infection or PJI by controlling for other confounding factors. This is related to the lower starting preoperative hemoglobin in SLE patients compared to that in the osteoarthritis population as reported [14, 33]. And Dorsch [34] also found that SLE is associated with abnormal platelet aggregation and anticoagulant factor antibodies, thus increasing the risk of perioperative bleeding. This may also explain why THA surgery in SLE patients increases DVT risk [35, 36].

This systematic review and meta-analysis have their limitations. Firstly, several kinds of literature with a large amount of data and high quality cannot obtain specific data in detail. Contacting the author is a pity that no reply has been given to get specific research results. Therefore, we cannot perform a subgroup analysis to see the incidence rate of complications. Secondly, most of the included studies are retrospective cohort studies, with small sample size and a moderate level of evidence. Thirdly, the specific reasons for the revision of some studies early were not reported. Therefore, we could not obtain further information about the specific causes of revision. Fourthly, the result of periprosthetic fractures in our study has heterogeneity. Thus, some bias may exit in our result. The reliability of the research results needs to be confirmed. Consequently, longer follow-up and higher-level studies are required to prove our conclusions.

## Conclusion

SLE Patients receiving THA are at an increased risk of DVT, wound infection, dislocation, periprosthetic fracture, revision, and periprosthetic joint infection in comparison non-SLE patients. There was no significant difference in HHS scores between SLE patients and non-SLE patients. Adequately powered studies, which incorporate control for appropriate confounders and other covariates, are needed to confirm these findings. The results of such studies would be helpful to guide decision-making regarding THA in the setting of SLE.

## Supplementary Information

**Additional file 1**. Search strategy of Pubmed.

## Data Availability

The authors declare that all the data supporting the findings of this study are available within the article and its supplementary information files.

## Abbreviations

SLE: Systemic lupus erythematosus
THA: Total hip arthroplasty
DVT: Deep vein thrombosis
HSS: Harris hip scores
ONFH: Osteonecrosis of the femoral head

## References

[1] Kwon HH, Bang SY, Won S, Park Y, Yi JH, Joo YB, Lee HS, Bae SC (2018). Synergistic effect of cumulative corticosteroid dose and immunosuppressants on avascular necrosis in patients with systemic lupus erythematosus. Lupus.

[2] Hussein S, Suitner M, Beland-Bonenfant S, Baril-Dionne A, Vandermeer B, Santesso N, Keeling S, Pope JE, Fifi-Mah A, Bourre-Tessier J (2018). Monitoring of osteonecrosis in systemic lupus erythematosus: a systematic review and metaanalysis. J Rheumatol.

[3] Kang Y (2013). Zhang Z-j, Zhao X-y, Zhang Z-q, Sheng P-y, Liao W-m: Total hip arthroplasty for vascular necrosis of the femoral head in patients with systemic lupus erythematosus: a midterm follow-up study of 28 hips in 24 patients. Eur J Orthopaedic Surg Traumatol.

[4] Domsic RT, Lingala B, Krishnan E (2010). Systemic lupus erythematosus, rheumatoid arthritis, and postarthroplasty mortality: a cross-sectional analysis from the nationwide inpatient sample. J Rheumatol.

[5] Hart A, Janz V, Trousdale RT, Sierra RJ, Berry DJ, Abdel MP (2019). Long-term survivorship of total hip arthroplasty with highly cross-linked polyethylene for osteonecrosis. J Bone Joint Surg-Am.

[6] Ehmke TA, Cherian JJ, Wu ES, Jauregui JJ, Banerjee S, Mont MA (2014). Treatment of osteonecrosis in systemic lupus erythematosus: a review. Curr Rheumatol Rep.

[7] Chen CH, Hsu CW, Lu MC (2019). Risk of joint replacement surgery in Taiwanese female adults with systemic lupus erythematosus: a population-based cohort study. BMC Musculoskelet Disord.

[8] Li Z, Du Y, Xiang S, Feng B, Bian Y, Qian W, Jin J, Lin J, Weng X (2019). Risk factors of perioperative complications and transfusion following total hip arthroplasty in systemic lupus erythematosus patients. Lupus.

[9] Singh JA, Cleveland JD (2019). Lupus is associated with poorer outcomes after primary total hip arthroplasty. Lupus.

[10] Aziz KT, Best MJ, Skolasky RL, Ponnusamy KE, Sterling RS, Khanuja HS (2020). Lupus and perioperative complications in elective primary total hip or knee arthroplasty. Clin Orthop Surg.

[11] Chen CH, Chen TH, Lin YS, Chen DW, Sun CC, Kuo LT, Shao SC (2020). The impact of systemic lupus erythematosus on the risk of infection after total hip arthroplasty: a nationwide population-based matched cohort study. Arthritis Res Ther.

[12] Kouk S, Baron SL, Pham H, Campbell A, Begly J, Youm T (2020). Clinical outcomes of hip arthroscopy in patients with systemic inflammatory diseases compared with matched controls at a minimum of 2-year follow-up. Arthroscopy - J Arthroscopic Relat Surg.

[13] Wang CC, Huang Y, Huang YD (2020). Systemic lupus erythematosus induced by adjuvants after metal-on-polyethylene total hip arthroplasty. Chin Med J.

[14] Cai Y, Ding Z, Rong X, Zhou ZK (2021). Does systemic lupus erythematosus increase the risk of complications from total hip arthroplasty?. BMC Musculoskelet Disord.

[15] Gu J, Zhang S, Chen L, Feng X, Li H, Feng H, Zhang L, Zhou Y (2021). Performing a safe and effective total hip arthroplasty on patients with inactive or stably active systemic lupus erythematosus with osteonecrosis. J Am Acad Orthop Surg.

[16] Kushnareva I, Makarov M, Popkova T, Khramov A, Maglevaniy S (2021). A retrospective study of perioperative management of patients with SLE in total hip arthroplasty. Ann Rheum Dis.

[17] Schnaser EA, Browne JA, Padgett DE, Figgie MP, D'Apuzzo MR (2016). Perioperative complications in patients with inflammatory arthropathy undergoing total hip arthroplasty. J Arthroplasty.

[18] Woo MS, Kang JS, Moon KH (2014). Outcome of total hip arthroplasty for avascular necrosis of the femoral head in systemic lupus erythematosus. J Arthroplasty.

[19] Taylor-Williams O, Nossent J, Inderjeeth CA (2020). Incidence and complication rates for total hip arthroplasty in rheumatoid arthritis: a systematic review and meta-analysis across four decades. Rheumatol Ther.

[20] Page MJ, McKenzie JE, Bossuyt PM, Boutron I, Hoffmann TC, Mulrow CD, Shamseer L, Tetzlaff JM, Akl EA, Brennan SE et al. The PRISMA 2020 statement: an updated guideline for reporting systematic reviews. BMJ 2021;372:n71 10.1136/bmj.n71 PMC8005924 33782057

[21] Stang A (2010). Critical evaluation of the Newcastle-Ottawa scale for the assessment of the quality of nonrandomized studies in meta-analyses. Eur J Epidemiol.

[22] Higgins JP, Thompson SG, Deeks JJ, Altman DG (2003). Measuring inconsistency in meta-analyses. BMJ.

[23] Merayo-Chalico J, Gonzalez-Contreras M, Ortiz-Hernandez R, Alcocer-Varela J, Marcial D, Gomez-Martin D (2017). Total Hip arthroplasty outcomes: an 18-year experience in a single center: Is systemic lupus erythematosus a potential risk factor for adverse outcomes?. J Arthroplasty.

[24] Roberts JE, Mandl LA, Su EP, Mayman DJ, Figgie MP, Fein AW, Lee YY, Shah U, Goodman SM (2016). Patients with systemic lupus erythematosus have increased risk of short-term adverse events after total hip arthroplasty. J Rheumatol.

[25] Issa K, Naziri Q, Rasquinha VJ, Tatevossian T, Kapadia BH, Mont MA (2013). Outcomes of primary total hip arthroplasty in systemic lupus erythematosus with a proximally-coated cementless stem. J Arthroplasty.

[26] Hussein S, Suitner M, Béland-Bonenfant S, Baril-Dionne A, Vandermeer B, Santesso N, Keeling S, Pope JE, Fifi-Mah A, Bourré-Tessier J (2018). Monitoring of osteonecrosis in systemic lupus erythematosus: a systematic review and metaanalysis. J Rheumatol.

[27] Abu-Shakra M, Buskila D, Shoenfeld Y (2003). Osteonecrosis in patients with SLE. Clin Rev Allergy Immunol.

[28] Fein AW, Figgie CA, Dodds TR, Wright-Chisem J, Parks ML, Mandl LA, Su EP, Salmon JE, Mayman DJ, Lee YY (2016). Systemic lupus erythematosus does not increase risk of adverse events in the first 6 months after total knee arthroplasty. J Clin Rheumatol: Pract Rep Rheum Musculoskeletal Diseases.

[29] Mertelsmann-Voss C, Lyman S, Pan TJ, Goodman S, Figgie MP, Mandl LA (2014). Arthroplasty rates are increased among US patients with systemic lupus erythematosus: 1991–2005. J Rheumatol.

[30] Chen DQ, Cancienne JM, Werner BC, Cui Q (2018). Is osteonecrosis due to systemic lupus erythematosus associated with increased risk of complications following total hip arthroplasty?. Int Orthop.

[31] Johannson HR, Zywiel MG, Marker DR, Jones LC, McGrath MS, Mont MA (2011). Osteonecrosis is not a predictor of poor outcomes in primary total hip arthroplasty: a systematic literature review. Int Orthop.

[32] Kang Y, Zhang ZJ, Zhao XY, Zhang ZQ, Sheng PY, Liao WM (2013). Total hip arthroplasty for vascular necrosis of the femoral head in patients with systemic lupus erythematosus: a midterm follow-up study of 28 hips in 24 patients. Eur J Orthop Surg Traumatol.

[33] Merayo-Chalico J, Gónzalez-Contreras M, Ortíz-Hernández R, Alcocer-Varela J, Marcial D, Gómez-Martín D (2017). Total hip arthroplasty outcomes: an 18-year experience in a single center: Is systemic lupus erythematosus a potential risk factor for adverse outcomes?. J Arthroplasty.

[34] Dorsch CA, Meyerhoff J (1982). Mechanisms of abnormal platelet aggregation in systemic lupus erythematosus. Arthritis Rheum.

[35] Linge P, Fortin PR, Lood C, Bengtsson AA, Boilard E (2018). The non-haemostatic role of platelets in systemic lupus erythematosus. Nat Rev Rheumatol.

[36] Takahashi H, Moroi M (2001). Antibody against platelet membrane glycoprotein VI in a patient with systemic lupus erythematosus. Am J Hematol.

